# PPTFH: Robust Local Descriptor Based on Point-Pair Transformation Features for 3D Surface Matching

**DOI:** 10.3390/s21093229

**Published:** 2021-05-07

**Authors:** Lang Wu, Kai Zhong, Zhongwei Li, Ming Zhou, Hongbin Hu, Congjun Wang, Yusheng Shi

**Affiliations:** 1 State Key Laboratory of Materials Processing and Die & Mould Technology, School of Materials Science and Engineering, Huazhong University of Science and Technology, Wuhan 430074, China; langwu@hust.edu.cn (L.W.); zwli@hust.edu.cn (Z.L.); walden@hust.edu.cn (C.W.); shiyusheng@hust.edu.cn (Y.S.); 2 Hubei Tri-Ring Forging Co., Ltd., Xiangyang 441700, China; zhouming@tring.cn (M.Z.); huhongbin@triring.cn (H.H.)

**Keywords:** local surface descriptor, 3D surface matching, object recognition, 3D registration

## Abstract

Three-dimensional feature description for a local surface is a core technology in 3D computer vision. Existing descriptors perform poorly in terms of distinctiveness and robustness owing to noise, mesh decimation, clutter, and occlusion in real scenes. In this paper, we propose a 3D local surface descriptor using point-pair transformation feature histograms (PPTFHs) to address these challenges. The generation process of the PPTFH descriptor consists of three steps. First, a simple but efficient strategy is introduced to partition the point-pair sets on the local surface into four subsets. Then, three feature histograms corresponding to each point-pair subset are generated by the point-pair transformation features, which are computed using the proposed Darboux frame. Finally, all the feature histograms of the four subsets are concatenated into a vector to generate the overall PPTFH descriptor. The performance of the PPTFH descriptor is evaluated on several popular benchmark datasets, and the results demonstrate that the PPTFH descriptor achieves superior performance in terms of descriptiveness and robustness compared with state-of-the-art algorithms. The benefits of the PPTFH descriptor for 3D surface matching are demonstrated by the results obtained from five benchmark datasets.

## 1. Introduction

Local surface description is a fundamental technology in 3D computer vision and robotics. It has been used in several applications, such as 3D point clouds registration [1,2,3,4,5,6], 3D shape retrieval [7,8], 3D object recognition [9,10], and robot manipulation [11,12,13]. A local surface descriptor is represented by a high-dimensional feature vector that encodes geometric and spatial information on a local surface. As we focus on local surface description for rigid objects, the local descriptor should be invariant to pose transformations. Moreover, owing to the existence of noise, mesh resolution variation, clutter, and occlusion in several applications, a local feature descriptor should exhibit strong robustness to resist the negative effects of these factors, and high descriptiveness to distinguish local surfaces. Thus, designing a local surface descriptor with high overall performance is a considerable challenge, and several attempts have been made to overcome the related difficulties. Depending on whether a local reference frame (LRF) is used, these descriptors are classified into two categories [14].

Regarding descriptors without LRF, numerous highly effective methods have been proposed [15], and descriptors based on point-pair features (PPFs) are the most classical methods in 3D surface description [16]. Johnson et al. [17] presented a spin-image (SI) local descriptor. This algorithm calculates two spatial distances using the key point—its normal and a neighbor point—then, the SI descriptor is generated by considering the distribution information of neighbor points along the two spatial features. This descriptor is very low in terms of time consumption, but it has weak descriptive performance owing to the poor surface information encoded by the two simple features [15]. Rusu et al. [18] proposed a point feature histogram (PFH) to describe a local surface for point cloud registration. The PFH is constructed by using several point-pair features that are computed using Darboux frames [19], defined at a point-pair. To reduce time consumption, a modified version of the PFH was proposed in [20]; that is, the fast PFH (FPFH) descriptor which is constructed by weighting the simplified PFH associated with all neighbor points. These descriptors only make use of single geometric or spatial features to encode surface information, resulting in poor performance in terms of descriptiveness under the effect of various disturbances (noise, clutter, occlusion, mesh decimation, etc.). Moreover, after analyzing the descriptive power of the four features that are used in the classical object recognition algorithm (i.e., PPF [21]), Buch et al. [22] proposed the point-pair feature histogram (PPFH) descriptor based on the two most discriminative features in PPF. Although this descriptor is highly efficient and descriptive, it is sensitive to noise and mesh resolution variations [16,23].

Regarding LRF-based methods, these descriptors can encode both geometric and spatial information on a local surface, according to the established LRFs. The best-known examples are based on a signature of histograms of orientations (SHOT) [24], rotational projection statistics (RoPS) [25], and triple orthogonal local depth images (TOLDI) [26]. The SHOT descriptor performs covariance matrix analysis to define the LRF and divides a spherical neighborhood space into 32 bins along the radial, azimuth, and elevation directions. It is constructed by considering point distribution information in 32 bins using the deviation angles between the key point normal and the neighbor normal. Despite its high descriptiveness, SHOT is sensitive to mesh resolution variations. The LRF of the RoPS descriptor is generated by the weighted scatter matrix on triangular meshes, and the RoPS descriptive algorithm is obtained by extracting a set of statistics from several point-distribution matrices. The RoPS descriptor has been proven to have high descriptiveness [15]; however, it is highly time-consuming. With regard to the TOLDI descriptor, LRF is first constructed by calculating the normal of the key point and the weighted projection vectors of all the radius neighbors of the key point. Then, TOLDI uses three local depth images, corresponding to three coordinate planes, to further form the TOLDI descriptor. This descriptor is robust to clutter and occlusion, but it is not compact [27]. The descriptiveness and robustness of these LRF-based feature descriptors depend on the descriptive algorithms, and the performance of these methods is affected by the stability and repeatability of the constructed LRF. Unfortunately, LRFs generated on local surfaces tend to suffer from low stability and sign ambiguity, and a small error of the LRF may significantly change the generated local descriptor; this negatively affects the descriptor matching results [27].

Based on this analysis, existing descriptors either only extract single geometric and spatial information or include unstable geometric and spatial information encoded by the LRFs, resulting in low descriptiveness and weak robustness under the influence of disturbances [23]. To address these drawbacks, we propose a novel local descriptor using point-pair transformation feature histograms (PPTFHs) for discriminative and robust surface description. Specifically, the point-pair sets on a local surface are elaborately partitioned into four subsets using a simple but efficient strategy. Then, using four point-pair transformation features calculated by the proposed Darboux frame, the point-pair distribution information is encoded by PPTFHs to generate the proposed descriptor. A series of experiments on five public datasets representing different 3D application scenarios demonstrate that the proposed descriptor achieves superior performance compared with existing methods. The major contributions of this study are summarized as follows.

1. A novel local surface descriptor (PPTFH) is proposed to achieve superior performance in terms of descriptiveness and robustness under various disturbances.
2. A simple but effective point-pair set partition strategy is introduced. It exhibits high repeatability and stability, and it can be applied to other PPF descriptors (e.g., PFH) to enhance their feature matching performance (Section 3).

The remainder of this paper is organized as follows. In Section 2, we describe in detail the generation process of the PPTFH descriptor. In Section 3, we present a performance evaluation of the PPTFH descriptor and other state-of-the-art algorithms on four popular datasets. In Section 4, we apply the proposed descriptor in 3D surface matching on five different application scenarios. The paper is concluded in Section 5.

## 2. Local Surface Description Based on PPTFHs

In general, a unique high-dimensional feature vector is used to describe the local 3D surface of a 3D key point in the field of 3D surface description. In our study, we propose point-pair transformation feature histograms (PPTFH) to describe a 3D local surface. The entire generation process of the PPTFH descriptor mainly consists of three steps. First, local point-pair sets are divided into four subsets through a simple but stable spatial cue. Subsequently, three 2D histograms are constructed using the computed point-pair transformation features on each point-pair subset. Finally, all feature histograms corresponding to the four subsets are concatenated into a feature vector to represent the PPTFH descriptor. Furthermore, the four key parameters of the PPTFH descriptor are quantitatively analyzed.

### 2.1. Point-Pair Set Partition

The approach of tackling point-pairs on the local surface is critical for encoding rich surface information and constructing a descriptive and robust descriptor. Existing methods either do nothing for the point-pair sets or divide them into several subsets based on unstable geometric features [18,28], resulting in low descriptiveness and weak robustness. Hence, we propose a novel method to accurately partition point-pair sets into four subsets.

**Figure 1:**
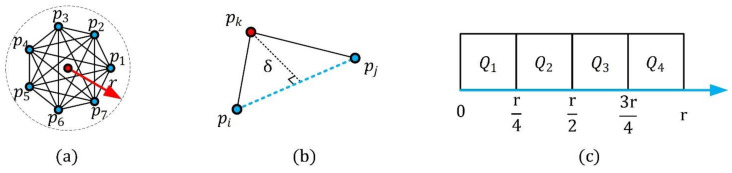
Partition of the point-pair sets. (**a**) All point-pairs in the key point neighborhood. (**b**) The partition feature δ of the point-pair $\left(p_{i},p_{j}\right)$. (**c**) The four point-pair subsets based on the feature δ.

For a key point $p_{k}$ and support radius r, its neighbors are obtained as $S_{p_{k}}=\left\{p_{i}:||p_{i}-p_{k}||\leq r\wedge p_{i}\neq p_{k},\;i\in \left\{0,1,\;\ldots ,n\right\}\right\}$, where n represents the number of the neighbors. It should be noted that the key point $p_{k}$ is not included in its neighbors. Then, the point-pair sets around $p_{k}$ are constructed as $Q_{p_{k}}=\{\left(p_{i},p_{j}\right)|p_{i},p_{j}\in S_{p_{k}}\wedge p_{i}\neq p_{j}\}$, as shown in Figure 1a. Concerning the key point $p_{k}$ and each point-pair $\left(p_{i},p_{j}\right)\in Q_{p_{k}}$, a spatial feature δ is computed, as shown in Figure 1b. It is the Euclidean distance from the key point $p_{k}$ to the straight line, determined by the point-pair $\left(p_{i},p_{j}\right)$, that is,

(1) $$\delta =\frac{||(p_{j}-p_{i})\times \left(p_{k}-p_{i}\right)||}{||p_{j}-p_{i}||}.$$

The value of δ is in the interval $\left[0,r\right]$. This feature δ is used to divide the point-pair sets $Q_{p_{k}}$ into $N_{\delta }$ regions, Herein, we set $N_{\delta }=4$ accord to the parameter analysis experiment result in Section 2.3; that is, the range of δ is equally partitioned into four parts, and, thus, we can obtain four sub-regions{$Q_{1},Q_{2},Q_{3},Q_{4}$}, as shown in Figure 1c. For instance, assume that $p_{1},p_{2}$ are the neighbors of the key point $p_{k}$, and the feature $\delta_{12}\in \left[3r/4,r\right]$, then, the point-pair $\left(p_{1},p_{2}\right)\in Q_{4}$. Accordingly, δ is able to elaborately and stably partition local point-pair sets.

### 2.2. Transformation Feature Histogram Generation

After the point-pair sets have been divided into four subsets, the point-pair trans-formation is calculated using the proposed Darboux frames, and the local descriptor is generated by the computation of the PPTFHs.

#### 2.2.1. Definition of a Novel Darboux Frame

**Figure 2:**
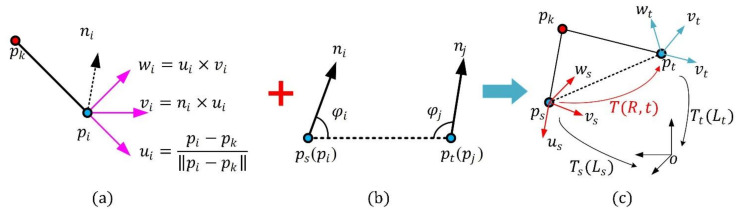
Generation of a point-pair transformation matrix. (**a**) Definition of proposed Darboux frame. (**b**) Definition of source point and target point. (**c**) Computation of point-pair transformation matrix.

An illustration of the proposed Darboux frame is shown in Figure 2a. For a key point $p_{k}$, a neighbor point $p_{i}$, and the normal $n_{i}$ of $p_{i}$, a new Darboux frame with its origin at $p_{i}$ can be represented as

(2) $$L_{i}=\left\{u_{i},v_{i},w_{i},p_{i}\right\}$$

where $u_{i}$ is equal to the normalized vector $\vec{p_{k}p_{i}}$, and $v_{i}$ is computed by the cross-product of $n_{i}$ and $u_{i}$. It is worth noting that the normal and the associated sign are estimated using the PCA (Principal Component Analysis) method presented in [28], with a support radius of 5 mr (mr denotes mesh resolution, which is the average distance between each point and its nearest neighbor point in 3D data). Then, $w_{i}$ is generated by the cross-product of $u_{i}$ and $v_{i}$, and $p_{i}$ is the origin point of the Darboux frame.

Compared with the Darboux frame mentioned in [19], the defined $u_{i}$ axis and novel Darboux frame can further increase the robustness of the proposed local descriptor. This is explained in Section 2.3.

#### 2.2.2. Point-Pair Transformation Feature Histogram Computation

First, a local point-pair set $Q_{p_{k}}$ is generated using the local point cloud determined by a key point $p_{k}$ and the support radius r. The point-pair set $Q_{p_{k}}$ is divided into four subsets by the method presented in Section 2.1; that is, $Q_{p_{k}}=\{Q_{1},Q_{2},Q_{3},Q_{4}$}. Then, three PPTFHs are computed for each subset. Finally, the PPTFH descriptor is constructed using these 2D histograms.

Assuming that $Q_{n}$ $\left(n=1,2,3,4\right)$ is a subset of the point-pair set $Q_{p_{k}}$ corresponding to the key point $p_{k}$. For arbitrary point-pair ($p_{i},\;p_{j}$) in $Q_{n}$, we first compute two angles $\varphi_{i}$ and $\varphi_{j}$ between the two normals $n_{i}$, $n_{j}$ and the line segment $\bar{p_{i}p_{j}}$. Then, the point with the smaller angle of $\varphi_{i}$ and $\varphi_{j}$ is defined as a source point $p_{s}$, and the other point is the target point $p_{t}$, as is shown in Figure 2b. We construct two Darboux frames $L_{s}$ and $L_{t}$, where $L_{s}$ is obtained using the key point $p_{k}$ and the source point $p_{s}$ by the method proposed in Section 2.2.1, and $L_{t}$ is obtained similarly. The results are as follows:

(3) $$L_{s}=\left\{u_{s},v_{s},w_{s},p_{s}\right\},\left(u_{s}=\frac{\vec{p_{k}p_{s}}}{||\vec{p_{k}p_{s}}||},\;v_{s}=n_{s}\times u_{s},\;w_{s}=u_{s}\times v_{s}\right),\;L_{t}=\left\{u_{t},v_{t},w_{t},p_{t}\right\},\;\left(u_{t}=\frac{\vec{p_{k}p_{t}}}{||\vec{p_{k}p_{t}}||},\;v_{t}=n_{t}\times u_{t},\;w_{t}=u_{t}\times v_{t}\right).$$

For a Darboux frame, a transformation matrix could be obtained by transferring the Darboux frame system to the base coordinate system (it is a 4 × 4 identity matrix mathematically) (see Figure 2c). Hence, with regard to the Darboux frames $L_{s}$ and $L_{t}$, we could obtain two transformation matrices $T_{s}$ and $T_{t}$ respectively. The two transformation matrices are defined as

(4) $$T_{s}=\left[\begin{array}{cc} R_{s} & p_{s}\\ 0 & 1 \end{array}\right],R_{s}=\left[u_{s},v_{s},w_{s}\right],\;T_{t}=\left[\begin{array}{cc} R_{t} & p_{t}\\ 0 & 1 \end{array}\right],R_{t}=\left[u_{t},v_{t},w_{t}\right],$$

The transformation matrix $T\left(R,t\right)$ from the $p_{s}$ Darboux frame to the $p_{t}$ Darboux frame can now be calculated using the transformation matrices $T_{t}$ and $T_{s}$ as follows:

(5) $$T=\left[\begin{array}{cc} R & t\\ 0 & 1 \end{array}\right]=T_{t}{}^{-1}T_{s}=\left[\begin{array}{cc} R_{t}{}^{T}R_{s} & R_{t}{}^{T}\left(p_{s}-p_{t}\right)\\ 0 & 1 \end{array}\right]$$

where the rotation part R of T is computed using the following equation:

(6) $$R=\left[\begin{array}{c} u_{t}{}^{T}\\ v_{t}{}^{T}\\ w_{t}{}^{T} \end{array}\right]\left[u_{s},v_{s},w_{s}\right]=\left[\begin{array}{ccc} r_{11} & r_{12} & r_{13}\\ r_{21} & r_{22} & r_{23}\\ r_{31} & r_{32} & r_{33} \end{array}\right].$$

Subsequently, the distance d between $p_{s}$ and $p_{t}$ is computed using the translation part t to indicate the relative position of the two points; moreover, the three Euler angles $\left(\alpha ,\beta ,\gamma \right)$ are calculated using the rotation part R to represent the pose relationship between the two normals. The detailed results are as follows:

(7) $$\left\{\begin{array}{l} d=||t||=||t||=||R_{t}{}^{T}\left(p_{s}-p_{t}\right)||=||p_{t}-p_{s}||\\ \alpha =arctan\left(r_{21}/r_{11}\right)\\ \beta =arctan\left(-r_{31}/\sqrt{r_{32}{}^{2}+r_{33}{}^{2}}\right)\\ \gamma =arctan\left(r_{32}/r_{33}\right) \end{array}\right..$$

Then, the four point-pair transformation attributes of a key point and a neighbor point-pair are

(8) $$\left\{\begin{array}{l} f_{1}=d\in \left[0,\;2r\right]\\ f_{2}=\cos\left(\alpha +\pi /2\right)\in \left[-1,1\right]\\ f_{3}=\cos\left(\beta +\pi /2\right)\in \left[-1,1\right]\\ f_{4}=\cos\left(\gamma +\pi /2\right)\in \left[-1,1\right] \end{array}\right.$$

where the three angle attributes ($f_{2}$, $f_{3}$, $f_{4}$) are represented by the cosines of the corresponding angles. This can enhance the descriptive power of the descriptor, as demonstrated in [22,24].

After the above four transfer features are introduced, we compute the four attributes $\left(f_{1},f_{2},f_{3},f_{4}\right)$ for each point-pair in the partition $Q_{n}$. Subsequently, we use the four attributes to describe the local surface. Certain binning policies are presented in [19,20,25], and we use two-dimensional bins to encode the four-attribute information and achieve optimal description performance [28].

The number of bins for dividing the ranges of the distance attribute $f_{1}$ and the three angle attributes $f_{2}$, $f_{3}$, $f_{4}$ are, respectively, $N_{d}$ and $N_{a}$. The two parameters are determined by the parameter analysis experimental results in Section 2.3, and we discretize the three 2D attribute spaces (i.e.,$\;\left(f_{1},f_{2}\right)$,$\;\left(f_{1},f_{3}\right)$, $\left(f_{1},f_{4}\right)$) using $N_{d}\times N_{a}$ bins. For each 2D attribute space, a point-pair transformation feature histogram H is generated by counting the number of point-pairs in the partition $Q_{n}$ that fall into each 2D grid. The histogram H represents the distribution information of all point-pairs in the region $Q_{n}$.

Moreover, the performance of normal-based surface descriptors is affected by, for example, Gaussian noise and variable mesh resolution; hence, to reduce sensitivity to point density variations and noise, each histogram is interpolated bi-linearly and normalized to sum up to 1, and the PPTFH descriptor is constructed by concatenating all the histograms into a one-dimensional histogram (as in [29]). The length of the PPFTH surface descriptor is $4\times 3\times N_{d}\times N_{a}$.

In the following, we theoretically demonstrate the superior discriminability and robustness of the proposed descriptor. The merits of the PPFTH descriptor can be summarized in at least three aspects.

First, compared with the partition strategy presented in HoPPF[28], without relying on the unstable normal, a simple but stable spatial cue is introduced to robustly divide the point-pair sets into four subsets. The division strategy is capability of improving the descriptive power and robustness of the PPTFH descriptor [23]. Second, in contract to HoPPF, our PPTFH descriptor defines two new Darboux frames based on point-pair $\left(p_{s},p_{t}\right)$, their normals $\left(n_{s},n_{t}\right)$ and the key point $p_{k}$; then, the point-pair transformation matrix is computed using the two defined Darboux frames in Section 2.2.1. The point-pair transformation matrix calculated by our method not only encodes the point-pair relative position and their normals relative rotation information, but also implies the angle information between the $\vec{p_{k}p_{s}}$ and $\vec{p_{k}p_{t}}$. The above facts indicate that our point-pair transformation matrix implies more rich and stable local point-pair information, thus improving the descriptiveness of our PPTFH descriptor and robustness to nuisances [23]. Finally, the point-pair distribution histograms are interpolated bi-linearly and normalized, ensuring resistance to point density variations and Gaussian noise.

### 2.3. Parameter Analysis for PPTFH Descriptor

**Figure 3:**
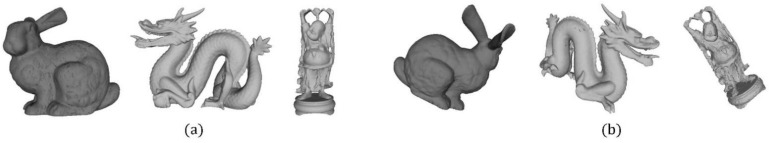
Examples on tuning dataset. (**a**) The examples of some models. (**b**) The examples of some scenes with 0.5 mr Gaussian noise and resampling 1/4 of model resolution.

In most local surface descriptors [14], the neighborhood radius is a common and critical parameter. To achieve a balance between descriptiveness, robustness, and time efficiency, we set the support radius to r=15 mr in all performance evaluation experiments, according to the suggestion presented in [26]. In addition to the support radius, four other parameters should be determined in the PPFTH method: different computing methods of the point-pair transformation matrix, the point-pair sets partition number $N_{\delta }$, the bin number $N_{d}$ of the Euclidean distance feature, and the partition number $N_{a}$ corresponding to three angle features. To determine reasonable parameter configurations, we use the classical recall versus 1-precision curve (RPC) criterion [26] to quantitatively evaluate the performance of the PPFTH descriptor for different parameter sets on the tuning dataset. The tuning dataset in this experiment includes 12 range images with 6 models and 6 scenes. The model range images are from the Bologna Retrieval dataset [24], and the scene data can be obtained by rotating the models, resampling the models to 1/4 of their original mesh resolution, and adding Gaussian noise, with a standard deviation of 0.5 mr. Examples of the models and scene images are shown in Figure 3.

**Figure 4:**
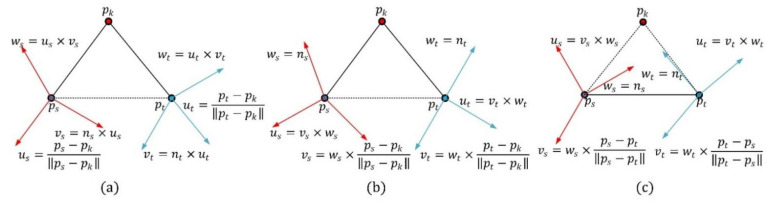
Different computing methods of the point-pair transformation matrix. (**a**) Proposed method. (**b**) ***Method-1***. (**c**) ***Method-2***.

**Figure 5:**
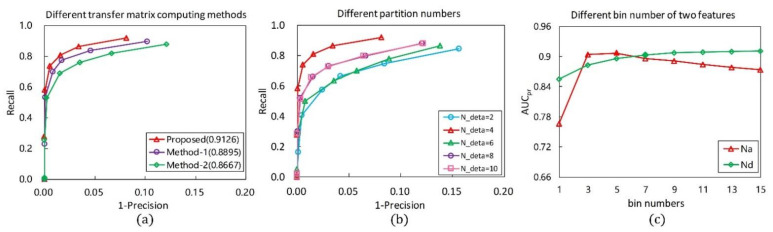
RPC and $AUC_{pr}$ results with different parameter configurations. (**a**) Different methods for computing point-pair transformation matrix, and the other parameters are separately set as r=15 mr,$\;N_{\sigma }=4$, $N_{a}=5$, $N_{d}=7$ (the values in parentheses are the $AUC_{pr}$ results). (**b**) Different point-pair set partition numbers. (**c**) Different bin numbers of two types of features.

The computing method of the point-pair transformation matrix is critical for the construction of the PPFTH descriptor. Different computing methods of the point-pair transformation matrix have a strong effect on the performance of the descriptor. In addition to the proposed computing method in Figure 4a, depending on whether the key point is used or not, there are two methods to calculate the point-pair transformation matrix, as shown in Figure 4b,c. One method is that the transformation matrix corresponding to each point-pair is computed using the two Darboux frames, each of which is defined by combining the connecting line between one neighbor point and the key point, with the neighbor point normal (Figure 4b). The other makes use of the two Darboux frames which are constructed by combining the line determined by the point-pair with their normals, as in [28] (Figure 4c). The two methods are referred to as ***Method-1*** (Figure 4b) and ***Method-2*** (Figure 4c). We used the two methods in place of the proposed methodology, whereas the other parameters were set to be constant. The obtained RPCs are shown in Figure 5a, and it is clearly seen that the PPTFH descriptor with the proposed method achieved the best performance, followed by the PPTFH with ***Method-1*** and ***Method-2***. The superior performance of the proposed method is because the Euler angles computed by the proposed method not only encode the relationship between two neighbor points, but also include the angle deviation information of the two connecting lines between the two neighbor points and the key point. Consequently, the proposed method was selected to compute the point-pair transformation matrix.

The point-pair sets partition number $N_{\sigma }$ is a critical parameter which affects the descriptiveness, time efficiency, and compactness of the PPTFH descriptor. Figure 5b presents the RPC results under different partition numbers in the tuning dataset. Obviously, the best performance is achieved when partition number $N_{\sigma }$ is equal to 4. The other two important parameters are the bin numbers $N_{d}$ and $N_{a}$. The configuration of these parameters greatly affects the robustness and discriminability of the proposed method. Hence, we test the performance of the descriptor for different bin numbers. The RPC results are presented in Figure 5c, where it can be observed that the best performance is achieved when the partition numbers of the Euclidean distance and angle features are set to 7 and 5, respectively. Accordingly, we eventually set $N_{d}=7$, $N_{a}=5$ in this study, and the dimensionality of the PPTFH descriptor is equal to 4×3×7×5=420.

## 3. Performance Evaluation Experiments

Herein, we test the performance of the PPTFH descriptor in various application scenarios using RPC (Recall Precision Curve) and ${\mathrm{AUC}}_{pr}$ (Area Under Curve) as evaluation metrics [26]. We first describe the implementation details of the experiments, namely the benchmark datasets, the compared methods, and evaluation metrics. Then, the proposed PPTFH descriptor is compared with state-of-the-art surface descriptors in terms of descriptiveness, robustness, compactness and time efficiency. Moreover, the generalization ability of the proposed point-pair division strategy is verified by the experimental results. All the experiments were implemented in VS2017 and PCL and conducted on a PC with an Intel Core i7-8700 3.2 GHz CPU and 16 GB of RAM.

### 3.1. Experiment Datasets and Methods

**Figure 6:**
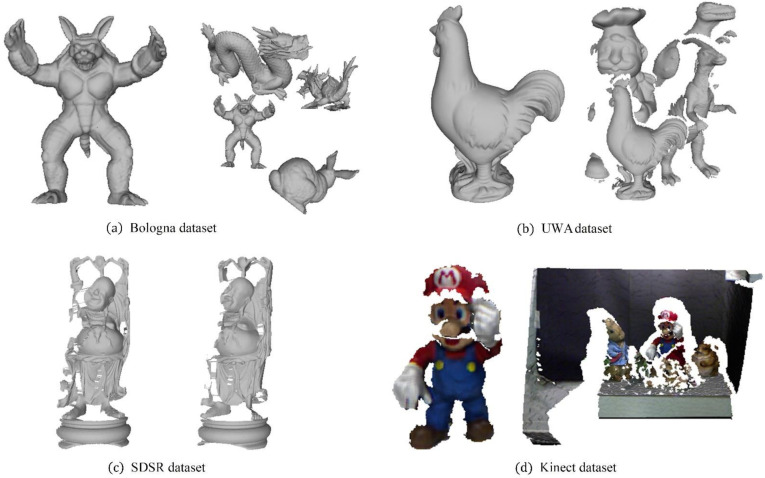
Examples of 4 models and scenes on 4 datasets. (**a**) Bologna dataset. (**b**) UWA dataset. (**c**) SDSR dataset. (**d**) Kinect dataset.

Four popular datasets were selected to conduct a series of experiments: the Bologna retrieval (BR) dataset [24] for 3D shape retrieval, the Stanford 3D scanning repository (SDSR) dataset [30] for partial 3D data registration, the UWA dataset [31] for 3D object recognition, and the Kinect dataset [32] for 3D object recognition with low-quality surfaces. Examples are shown in Figure 6.

More specifically, there are 6 3D models and 45 synthetic scenes in the BR dataset. To evaluate robustness, we resample all scenes to 1/2, 1/4, 1/8, and 1/16 of the original mesh resolution to enhance the nuisance factors in this dataset, and add Gaussian noise, with a standard deviation of 0.1, 0.3, 0.5, 0.7, and 0.9 mr, separately to the ¼-mesh-resolution scenes. The UWA dataset involves 5 models and 50 real scenes that are generated by scanning several real objects with random placement. Consequently, clutter and occlusions are the main challenges in this dataset. The SDSR dataset separately contains 15 scans from Happy-Buddha and 15 scans from Dragon. The nuisance factors in this dataset are missing regions, holes, and self-occlusions. The Kinect dataset consists of 6 models and 16 scenes acquired by the Microsoft Kinect sensor. In addition to the low mesh quality, moderate occlusion and clutter are also nuisance factors in this dataset.

In all comparative experiments, the PPTFH descriptor was compared with the most representative methods for performance evaluation in different 3D vision applications. These descriptors were divided into two categories: PPF-based and LRF-based methods. The PPF-based descriptors included PFH [18], FPFH [20], PPFH [22] and HoPPF [28], all of which were generated using a variety of point-pair features. In addition, we selected two well-known LRF-based descriptors (i.e., SHOT [24] and TOLDI [26]) to be compared with the proposed descriptor. In addition, to evaluate the generalization ability of the proposed point-pair set division method, we applied this strategy to the PFH descriptor. Specifically, the point-pair sets were partitioned into 4 subsets and the PFH descriptor in each subset was computed as a sub-feature. Then, the modified PFH (MoPFH) descriptor was generated by concatenating the four sub-features into a vector, and the dimensions of the MoPFH were 4×125=500. The parameter information of these state-of-the-art descriptors is listed in Table 1.

It should be noted that, in all experiments, using the uniform sampling method mentioned in [14], we sampled around 1000 points as the key points in each model to compute the descriptors. The key points in the scenes were obtained by transferring the model key points to the scene using the ground truth given by the four datasets. We uniformly set the support radius of all comparative descriptors to 15 mr to ensure fairness in the comparisons.

### 3.2. Evaluation Metrics

In order to quantitatively assess the descriptiveness, robustness and compactness, we adapted the popular Recall vs. 1-Percision Curve (RPC) and Area Under Curve (${\mathrm{AUC}}_{pr}$) as performance evaluation metrics. Note that ${\mathrm{AUC}}_{pr}$ is the area between the RPC and the 1-precision axis. The RPC results can be generated through the following steps.

First, for giving a model, a scene, and the ground truth pose, we randomly sampled around 1000 points as the key points in each model, and the key points in each scene could be obtained through translating the model key points to the scene using the ground true transformation matrix. Then, the descriptor of each model key point was matched with all scene key points descriptors to search the closest and the second closest descriptor. If the ratio ε between the closest and the second closest descriptor distances was smaller than a given threshold τ, the model key point and the scene key point with the closest descriptor distance would be considered a match. A match would be further defined as a correct one if the Euclidean distance between the transformed model key point and the scene key point was sufficiently small (i.e., being smaller than $\frac{1}{3}$ of the support radius of the descriptor in this study), otherwise it was regarded as a false match. Consequently, in a certain threshold, the recall and 1-percision are separately defined as:

(9) $$\mathrm{recall}=\frac{\mathrm{the}\;\mathrm{number}\;\mathrm{of}\;\mathrm{correct}\;\mathrm{matches}}{\mathrm{total}\;\mathrm{number}\;\mathrm{of}\;\mathrm{corresponding}\;\mathrm{descriptors}}\;1-\mathrm{percision}=\frac{\mathrm{the}\;\mathrm{number}\;\mathrm{of}\;\mathrm{false}\;\mathrm{matches}}{\mathrm{total}\;\mathrm{number}\;\mathrm{of}\;\mathrm{matches}}$$

Finally, the RPC result would be generated through setting a series of threshold. In our study, the series of thresholds for calculating the RPC were set as 0.3, 0.4, 0.6, 0.75, 0.85, 0.9, 0.95, 1.0, respectively. It is worth noting that the RPC result will locate at the upper left areas when the descriptor match obtains both of high recall and precision.

### 3.3. Performance Evaluation Results and Discussion

**Figure 7:**
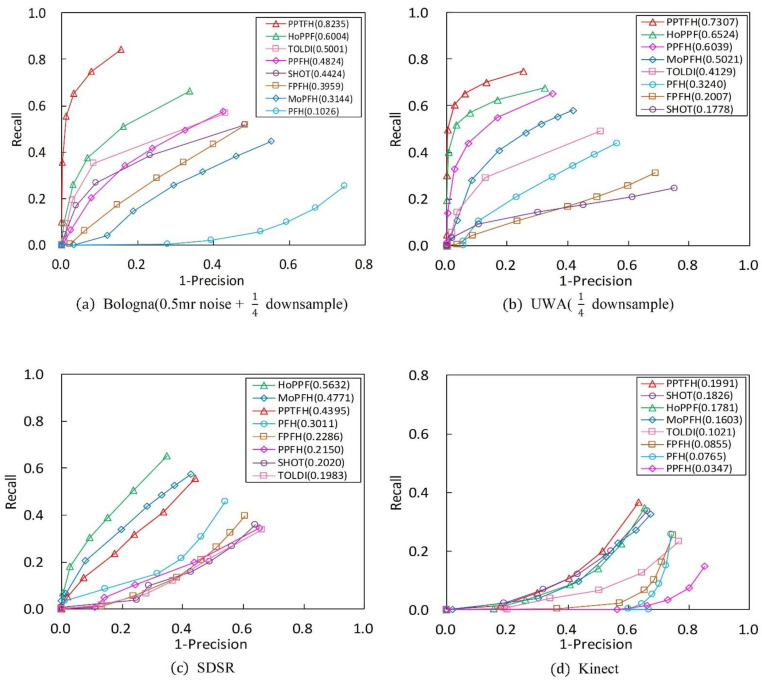
RPC results on 4 different application datasets (${\mathrm{AUC}}_{pr}$ values are exhibited in parentheses). (**a**) Bologna dataset with 0.5 mr noise and 1/4 downsample. (**b**) UWA dataset with 1/4 downsample. (**c**) SDSR dataset for registration. (**d**) Kinect dataset.

**Figure 8:**
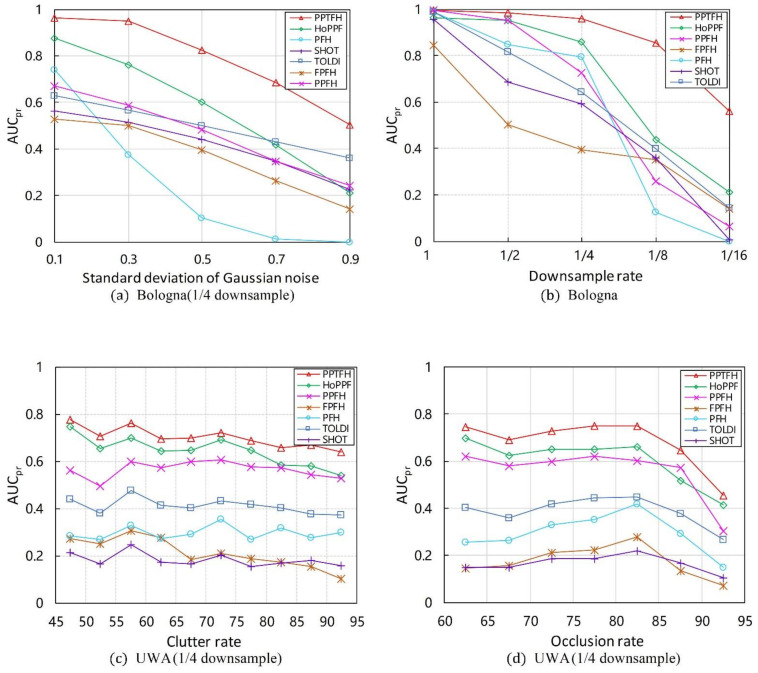
${\mathrm{AUC}}_{pr}$ results on different nuisances. (**a**) Bologna dataset with different noise levels and 1/4 downsample. (**b**) Bologna dataset with mesh resolution variation. (**c**) UWA dataset for different clutter rates. (**d**) UWA dataset for different occlusion rates.

The performance of the PPTFH descriptor is compared with that of the descriptors in Table 1 in terms of the RPC and ${\mathrm{AUC}}_{pr}$ metrics (Figure 7 and Figure 8) on the 4 benchmark datasets. The evaluation is in terms of local surface descriptiveness, robustness to various nuisance factors, compactness [14], and time efficiency. The details are as follows.

#### 3.3.1. Descriptiveness of the PPTFH Descriptor

As is shown in Table 2, our PPTFH descriptor is superior to the state-of-the-art methods in terms of descriptiveness. More specifically, according to the results in Figure 7a on the BR dataset with 0.5 mr noise and 1/4 decimation resolution, the proposed PPTFH method outperforms the other descriptors in terms of descriptiveness by a large margin (at least 0.2 regarding ${\mathrm{AUC}}_{pr}$), followed by HoPPF. Moreover, the HoPPF descriptor outperforms the other descriptors by 0.1 regarding the ${\mathrm{AUC}}_{pr}$ values, which is consistent with the results in [28]. The PFH descriptor is significantly inferior to the others because it is more sensitive to noise. Clutter and occlusion are the main challenges in the UWA dataset compared with the BR dataset. To improve computational efficiency, we resample the scenes with 1/4 mesh decimation, and the key points on the scene boundary are removed. The PRC and ${\mathrm{AUC}}_{pr}$ results for the UWA dataset are shown in Figure 7b. Evidently, the proposed PPTFH descriptor outperforms all the others by a large margin again, followed by the HoPPF and PPFH descriptors with similar performance. Moreover, compared with the three methods, the other descriptors exhibit a dramatic descriptive performance degradation on the UWA dataset. The SDSR dataset contains some 2.5D range images from different views, involving missing regions, holes, and self-occlusions. As shown in Figure 7c, the HoPPF descriptor achieves the best performance by a wide margin, followed by MoPFH and the proposed PPTFH method. Moreover, the other methods are inferior to the aforementioned three descriptors. In contrast to the above three datasets, the Kinect dataset is obtained by the cheap Kinect 3D sensor, and the mesh quality of the scanned range images is lower than that of 3D data scanned by a laser scanner. Therefore, in terms of PRC and ${\mathrm{AUC}}_{pr}$ values, the results in Figure 7d are inferior to those on the above three datasets by a large margin. Furthermore, the PPTFH descriptor is slightly superior to the SHOT method, followed by HoPPF. Remarkably, the descriptiveness of the PPFH descriptor is inferior to the other methods, and this observation coincides with the results in [28]. Moreover, from the results shown in Figure 7a–d, the modified PFH (MoPFH) descriptor with the proposed division strategy achieves a significant performance improvement compared with the original PFH. The results demonstrate that using the proposed point-pair set division strategy could enhance the performance of point-pair descriptors.

A comprehensive analysis of the results on the four datasets indicates two interesting phenomena. One is that the descriptiveness of early PPF-based methods (i.e., PFH and FPFH) is generally inferior to that of recent LRF-based descriptors (i.e., SHOT, TOLDI), which is because LRF-based methods can encode richer surface information than early PPF-based methods using the LRFs. Another shows that the most current PPF-based methods (i.e., the proposed PPTFH and HoPPF) outperform recent LRF-based methods in term of descriptive power, and it can be explained that the most current PPF-based descriptors (the proposed PPTFH and HoPPF) make full use of the spatial and geometric cues caused by the point-pair set partition strategy and novel point-pair features, and they are not affected by unstable LRFs.

#### 3.3.2. Robustness to Various Nuisance Factors

Herein, we use ${\mathrm{AUC}}_{pr}$ values to evaluate robustness of the PPTFH descriptor to Gaussian noise, mesh resolution variations, scene clutter, and occlusion. The experiments are only conducted on the Bologna and UWA datasets. The results are shown in Figure 8 and Table 3, Table 4 and Table 5.

Gaussian Noise. Regarding the robustness to Gaussian noise, we first resampled the Bologna dataset to 1/4 mesh resolution, and then added noise with a standard deviation of 0.1, 0.3, 0.5, 0.7, and 0.9 mr to each scene separately. The ${\mathrm{AUC}}_{pr}$ results for different levels of noise are shown in Figure 8a and Table 3. The proposed PPTFH descriptor achieved the best performance for each noise level, followed by the HoPPF and TOLDI descriptors. It can also be seen that the performance margin between the PPTFH descriptor and the other methods increased for high noise levels (i.e., 0.5, 0.7, and 0.9 mr Gaussian noise), and the PPTFH method achieved satisfactory performance, even at the highest noise levels (with an ${\mathrm{AUC}}_{pr}$ of at least 0.5, whereas the corresponding values for the other methods were less than 0.36).

Mesh Decimation. The proposed PPTFH descriptor performed the best at all levels of mesh decimation (Figure 8b and Table 3). More specifically, when the mesh decimation was low (i.e., 1/8 and even 1/16), the PPTFH method outperformed all the others by a large margin, and its ${\mathrm{AUC}}_{pr}$ values were always greater than 0.5, compared with those of the others, which were less than 0.25 for the lowest mesh decimation level (1/16).

Clutter and Occlusions. In the UWA dataset, the effects of scene clutter and occlusion on the performance of the PPTFH descriptor and the others were measured using the ${\mathrm{AUC}}_{pr}$ values, where the clutter rate was recomputed as suggested in [16] and the occlusion rate was provided by the UWA dataset. The results are shown in Figure 8c,d, and Table 4 and Table 5 for different levels of clutter and occlusion. In terms of robustness to clutter and occlusion, the PPTFH method outperformed the others for all clutter and occlusion levels, followed by the HoPPF and PPFH descriptors. Moreover, as the clutter rate increased, the performance of all descriptors did not change significantly, and the overall performance gradually decreased. The robustness to occlusion was consistent with that to clutter when the occlusion rate increased from approximately 60% to 80%; however, with an occlusion rate of more than 80%, the performance of all methods rapidly decreased because of the existence of boundary areas.

These results clearly demonstrate the strong robustness of the PPTFH descriptor to various nuisance factors (i.e., Gaussian noise and mesh decimation, scene clutter, and occlusion). Compared with the other surface descriptors, the proposed PPTFH method performs the best on both the Bologna retrieval dataset with noise and mesh resolution variation and the UWA object recognition dataset with clutter and occlusion. More importantly, satisfactory performance is also achieved by the PPTFH descriptor, even in extreme cases (0.9 mr noise and 1/16 mesh decimation).

#### 3.3.3. Compactness

In this part, the compactness of the state-of-the-art descriptors in Table 1 are evaluated on BR, UWA, SDSR and Kinect datasets. For a local descriptor, the compactness is also a significant attribute. It affects both the efficiency of feature matching and the size of memory usage. The compactness represents the performance of each floating-point number in a descriptor vector [14], which is defined as:

(10) $$\mathrm{Compactness}=\frac{{\mathrm{AUC}}_{pr}}{\mathrm{Dimensionality}}$$

where the ${\mathrm{AUC}}_{pr}$ values of the compared descriptors are presented in Figure 7 and Table 1 has given the dimensionality of these descriptors.

**Figure 9:**
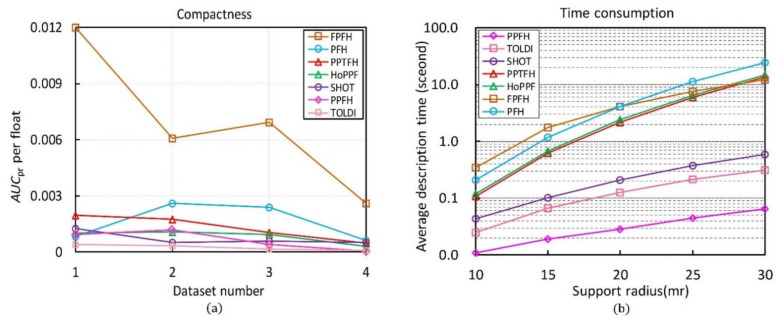
Evaluation results of compactness and time efficiency. (**a**) Compactness of all compared methods. (**b**) Time consumption of all compared methods, and the y axis is shown logarithmically for clarity.

The compactness of the compared descriptors calculated by the Equation (10) is shown in Figure 9a. The FPFH method achieves the best result in term of compactness, followed by the PFH. The high compactness of these two descriptors is mainly due to their very short lengths. Our PPTFH is the third compact descriptor owing to the high ${\mathrm{AUC}}_{pr}$ value, which means that our PPTFH achieves a balance between descriptiveness and compactness. Furthermore, the TOLDI method obtains a poor performance in term of compactness due to the long dimensionality of the TOLDI (up to 1200).

#### 3.3.4. Time Efficiency

In this section, we test the efficiencies of all the compared descriptors (see in Table 1). We first randomly sampled around 1000 key points in the tuning dataset (see in Section 2.3). Then, the total time costs of these descriptors generated on the extracted key points with different support radii (from 10 mr to 30 mr by increments of 5 mr) were counted, and the average consuming time for calculating one descriptor was considered as the final experiment result.

The evaluation results are shown in Figure 9b, some observations can be made. First, the PPFH achieves the optimal time efficiency, followed by the TOLDI and SHOT, because the time complexity of the three methods is $o\left(k\right)$ for the k-nearest neighbors compared with the other four methods (PPTFH, HoPPF, FPFH, PFH). Moreover, our PPTFH is moderate in terms of time efficiency, and it is a little more efficient than HoPPF due to the lower dimensionality (420 vs. 600). Finally, the PFH is the slowest descriptor because of its $o\left(k^{2}\right)$ computation complexity.

## 4. Applications to 3D Surface Matching

### 4.1. Surface Matching on Four Benchmark Datasets

To further validate the effectiveness of the PPTFH approach in different application scenarios, we applied the PPTFH descriptor and the aforementioned six methods (Table 1) to 3D correspondence-based surface matching on the BR, UWA, SDSR, and Kinect datasets. As in [22], the $F_{1}$ score was used to measure the surface matching performance of these local feature descriptors.

Specifically, we resampled the BR dataset for object retrieval to 1/4 mesh decimation and added Gaussian random noise with a standard deviation of 0.9 mr to the 1/4 mesh decimation scenes, and the UWA dataset and SDSR dataset were resampled to 1/4 mesh decimation. Each model was sampled to approximately 1000 points, as the model key points using the uniform sampling method mentioned in [14], and each scene was also sampled to appropriate numbers that could ensure sufficient key points for each instance in the scene. The local features corresponding to the key points were calculated by the local descriptors under comparison. Subsequently, the local features on model and scene served as input of the nearest neighbor distance ratio (NNDR) matching technique [33] to obtain the 3D correspondence points, and the 3D correspondence points were used as input to the surface-matching pipeline. Finally, to reject inappropriate 3D correspondence points and obtain a coarse pose estimation result, we adopted the geometric constraints (GC) in [34] to remove mismatch points, and the random sample consensus (RANSAC) [35] technique to estimate the pose transformation from the model to the scene.

It should be noted that, in all 3D surface matching experiments, the threshold in the NNDR method was set to 0.95, and the RANSAC iteration number was always equal to 10,000. Moreover, for each descriptor and the corresponding dataset, the other parameters of the matching pipeline were determined to maximize the number of true positives and minimize the number of false positives, so that the best performance in terms of the $F_{1}$ score might be achieved. With the other parameters remaining constant, we conducted a series of surface matching experiments under different support radii (increasing from 10 to 30 mr by increments of 5 mr).

**Figure 10:**
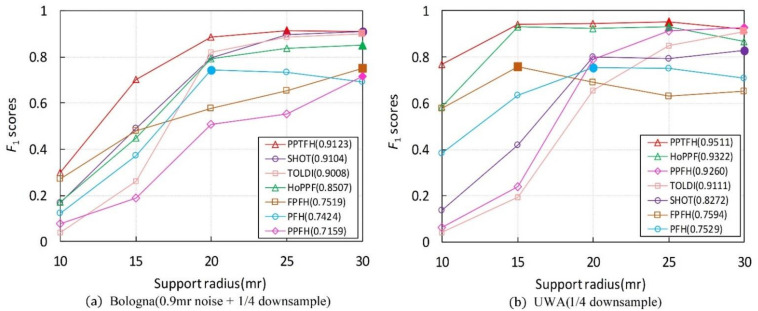

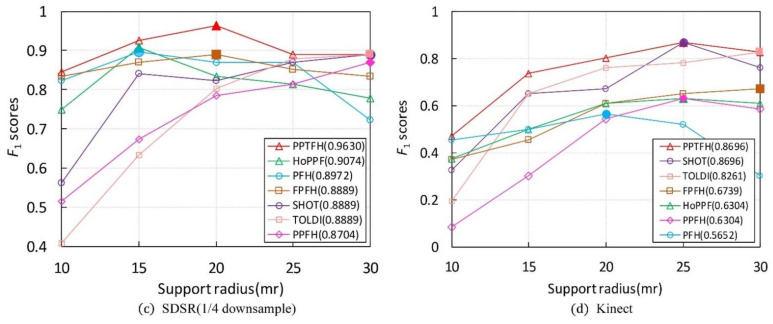
Surface matching performances ($F_{1}$ scores) under different support radiuses for different datasets. The position where the marker for solid color filling locates represents the support radius and $F_{1}$ scores when the performance of the descriptor achieves its best, and the value in parentheses represents the highest $F_{1}$ scores of each local descriptor. (Figure best seen in color).

The results are shown in Figure 10, and Table 6 lists the highest $F_{1}$ scores of each local descriptor. It can be observed that the proposed PPTFH descriptor achieves the best surface matching performance in all four datasets, followed by the HoPFF, TOLDI, and SHOT methods, which is consistent with the conclusions regarding the descriptive power in Section 3.3.1. Furthermore, the best performance is achieved by the proposed PPTFH method, even for different support radii. It can be concluded that, under noise, varying mesh resolution, clutter, occlusion, and mesh quality variations, the PPTFH method coherently provides a discriminative and robust description of the local surface.

In addition, as shown in Figure 10a, the matching performance of most descriptors (with the exception of the PFH) improves as the support radius increases. This is because, in this object retrieval dataset (BR dataset), the descriptors can encode more surface information and are not affected by clutter, occlusion, and missing mesh under a larger radius. However, Figure 10b–d indicates that the object recognition performance of most methods (except for PPFH and TOLDI) first improves and then degrades as the support radius increases. This can be explained by the fact that these descriptors could include richer surface features under a larger radius. However, the presence of clutter and occlusions, missing mesh, and boundaries in the three datasets has a seriously negative effect on the matching performance of the descriptors when the support radius is excessively large. Furthermore, from Figure 10, we can observe that different local descriptors have different support radii when the surface matching results achieve optimal performance on different datasets. This observation indicates that there is no constant support radius parameter to optimize the surface matching performance on different application scenarios and different nuisances (e.g., noise, clutter, occlusion, varying point density, and so on). In general, we recommend that it is enough that the support radius is selected from 15 mr to 25 mr in some applications, according to our study and other literature [14].

**Figure 11:**
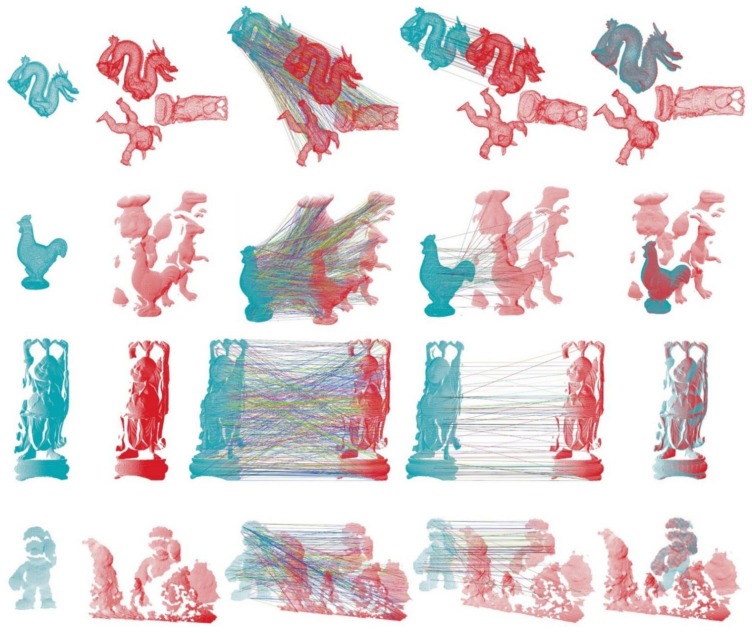
Sample visual registration results by our PPTFH descriptor on BR, UWA, SDSR, and Kinect datasets. From left to right: model point cloud (dark green color); scene point cloud (red color); correspondences by PPTFH+NNSR technology; correspondences by GC technology; and the registration result by RANSAC method.

We also present some visual surface matching results based on the our PPTFH descriptor, as shown in Figure 11. From the top down, these subfigures separately represent the surface matching visual sample on BR, UWA, SDSR, and Kinect datasets. From left to right, they represent the model, scene, correspondences obtained by using PPTFH descriptors matching and NNSR technology, correspondences after removing the mismatches by GC, and the surface matching results by RANSAC. One must note that the initial model and scene have obvious pose variation. After matching the PPTFH descriptors from scene to model, there are always enough correspondences to align the model to the scene by RANSAC transformation method.

### 4.2. Surface Matching on the WHU-TLS Dataset

Besides the surface matching on the above four benchmark datasets, we also applied our PPTFH descriptor to registration of the large-scale terrestrial laser scanner point clouds on the WHU-TLS dataset [3,4]. The WHU-TLS dataset consists of 115 scans from 11 different environments with varying point density, clutter, and occlusion. Herein, we selected 34 representative scene scans from 5 environments (i.e., mountain, campus, residence, riverbank, and heritage building) on the WHU-TLS dataset to perform the pairwise 3D registration experiment. More specifically, for a pair of point clouds, we first calculated the transformation matrix for registering the two-point clouds by using our proposed method. Then, if the error between the transformation matrix and the truth was less than the given threshold, we argued that the two-point clouds were registered correctly. The precision was the ratio of the correct registration number to the total pairs number.

**Figure 12:**
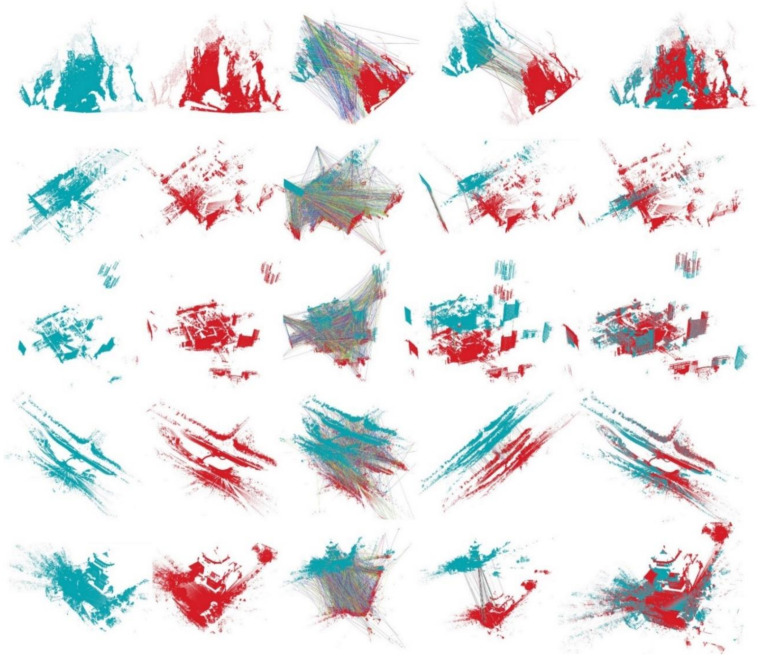
Sample visual registration results by our PPTFH descriptor on the WHU-TLS dataset. From left to right: model point cloud (dark green color); scene point cloud (red color); correspondences by PPTFH+NNSR technology; correspondences by GC technology; and the registration results by RANSAC method.

The registration experiment results are shown in Table 7. Despite the complex environment and low point cloud quality in these scans, we successfully achieve the 32 pairwise registration in 34 pair scans with a 94.12% precision rate. Moreover, some visual 3D registration results of the five environmental data are presented in Figure 12 (from top to bottom, mountain, campus, residence, riverbank, and heritage building), and we find that our proposed PPTFH descriptor is able to generate enough correspondences to align the two 3D point clouds.

## 5. Discussions and Conclusions

In this article, we proposed a novel PPTFH descriptor for 3D surface description, together with a proposed point-pair division strategy. The prominent advantage of our PPTFH descriptor is its high descriptiveness and strong robustness.

With regard to the point-pair division strategy, by computing the distance between the key point and the line determined by two neighbor points, a novel spatial feature was introduced to divide the point-pair sets into four subsets. Differing from the classical methods (i.e., PFH [18], FPFH [20]), which directly encode the surface information without tackling the point-pair sets, our technique utilizes a simple yet efficient spatial feature to divide the point-pair sets elaborately; thus, it can realize the improvement of the descriptiveness and robustness of the PPF-based descriptor.

The PPTFH descriptor was then generated with four sub-features, each of which was constructed by three transformation feature histograms corresponding to each point-pair subset. Both geometric and spatial information was encoded in the PPTFH descriptor in a comprehensive manner. The main characteristics of the proposed PPTFH descriptor are concluded as follows. First, the PPTFH descriptor make use of a stable and robust division strategy to preprocess the point-pair sets, which enhances the descriptiveness and robustness of the PPTFH. Furthermore, the PPTFH is highly informative because it makes use of four point-pair transformation attributes computed via our defined Darboux frames to encode the local surface. Finally, the PPTFH is robust to noise and various mesh resolutions, owing to the interpolation and normalization operations.

In order to evaluate our PPTFH descriptor, a series of experiments and comparisons were performed on the BR, UWA, SDSR, Kinect, and WHU-TLS datasets, which are, respectively, relevant to shape retrieval, object recognition, 3D registration, 3D object recognition with low-quality surfaces, and 3D terrestrial laser scanner point cloud registration in the domain of remote sensing. The result reveals that our proposed point-pair sets division strategy could be grafted to other point-pair-features-based methods (e.g., PFH) to improve descriptiveness and robustness, and the proposed PPTFH descriptor outperforms state-of-the-art methods by a large margin in terms of descriptiveness and robustness. In addition, the superior feature matching performance of the PPTFH descriptor is validated by its applications to 3D surface matching in five benchmark datasets.

In future, our work will focus on improving time efficiency, as time consumption of the PPTFH method is moderate when compared with the state-of-the-art methods. In addition, along with the development of RGB-D sensors, integrating color cues into our PPTFH descriptor is helpful for an application to objects that have limited geometric features but rich texture information.

## Figures and Tables

**Table 1:** Parameter settings for the state-of-the-art descriptors.

Method	Dimensionality	Length	Category ^1^
PFH	5 × 5 × 5	125	PPF
MoPFH	4 × 5 × 5 × 5	500	PPF
FPFH	3 × 11	33	PPF
PPFH	16 × 32	512	PPF
SHOT	8 × 2 × 2 × 11	352	LRF
TOLDI	3 × 20 × 20	1200	LRF
HoPPF	8 × 3 × 5 × 5	600	PPF
PPTFH	4 × 3 × 7 × 5	420	PPF

^1^ Category is the descriptor category. The PPF represents the point-pair features-based method, and the LRF represents the local reference frame-based method.

**Table 2:** Descriptiveness (${\mathrm{AUC}}_{pr}$) for different datasets.

Datasets	Bologna (0.5 mr Noise + 1/4 Downsample)	UWA (1/4 Downsample)	SDSR	Kinect
Method				
PFH	0.1026	0.3240	0.3011	0.0765
MoPFH	0.3144	0.5021	0.4771	0.1603
FPFH	0.3959	0.2007	0.2286	0.0855
PPFH	0.4824	0.6039	0.2150	0.0347
SHOT	0.4424	0.1778	0.2020	0.1826
TOLDI	0.5001	0.4129	0.1983	0.1021
HoPPF	0.6004	0.6524	**0.5632**	0.1781
PPTFH	**0.8235**	**0.7307**	0.4395	**0.1991**

The best ${\mathrm{AUC}}_{pr}$ results are in bold fonts.

**Table 3:** Robustness (${\mathrm{AUC}}_{pr}$) to different nuisances in Bologna dataset.

Nuisances	Noise (1/4 Downsample)					Downsample				
Method	0.1 mr	0.2 mr	0.5 mr	0.7 mr	0.9 mr	1	1/2	1/4	1/8	1/16
PFH	0.7418	0.3770	0.1026	0.0121	0.0008	0.9887	0.8465	0.7945	0.1241	0.0001
FPFH	0.5275	0.5023	0.3959	0.2639	0.1405	0.8443	0.5023	0.3959	0.3523	0.1405
PPFH	0.6719	0.5880	0.4824	0.3489	0.2442	0.9942	0.9507	0.7267	0.2578	0.0637
SHOT	0.5648	0.5139	0.4424	0.3483	0.2271	0.9566	0.6847	0.5916	0.3574	0.0078
TOLDI	0.6285	0.5680	0.5001	0.4293	0.3609	0.9884	0.8164	0.6440	0.3994	0.1414
HoPPF	0.8768	0.7602	0.6004	0.4157	0.2122	0.9606	0.9533	0.8598	0.4376	0.2122
PPTFH	**0.9650**	**0.9490**	**0.8235**	**0.6837**	**0.5030**	**0.9958**	**0.9826**	**0.9593**	**0.8561**	**0.5595**

The best ${\mathrm{AUC}}_{pr}$ results are in bold fonts.

**Table 4:** Robustness (${\mathrm{AUC}}_{pr}$) to clutter in UWA dataset (1/4 downsample).

Nuisances	Clutter									
Method	0.45–0.50	0.50–0.55	0.55–0.60	0.60–0.65	0.65–0.70	0.70–0.75	0.75–0.80	0.80–0.85	0.85–0.90	0.90–0.95
PFH	0.2833	0.2703	0.3265	0.2715	0.2919	0.3556	0.2675	0.3171	0.2780	0.2981
FPFH	0.2736	0.2492	0.3044	0.2765	0.1828	0.2088	0.1872	0.1740	0.1558	0.1040
PPFH	0.5623	0.4936	0.5991	0.5711	0.5968	0.6043	0.5775	0.5708	0.5415	0.5264
SHOT	0.2135	0.1651	0.2474	0.1711	0.1659	0.2024	0.1528	0.1695	0.1808	0.1569
TOLDI	0.4385	0.3806	0.4754	0.4120	0.4025	0.4329	0.4165	0.4012	0.3778	0.3729
HoPPF	0.7455	0.6555	0.6990	0.6424	0.6482	0.6899	0.6474	0.5845	0.5808	0.5388
PPTFH	**0.7752**	**0.7048**	**0.7631**	**0.6961**	**0.6979**	**0.7199**	**0.6883**	**0.6588**	**0.6705**	**0.6403**

The best ${\mathrm{AUC}}_{pr}$ results are in bold fonts.

**Table 5:** Robustness (${\mathrm{AUC}}_{pr}$) to occlusion in UWA dataset (1/4 downsample).

Nuisances	Occlusion						
Method	0.60–0.65	0.65–0.70	0.70–0.75	0.75–0.80	0.80–0.85	0.85–0.90	0.90–0.95
PFH	0.2564	0.2628	0.3317	0.3529	0.4181	0.2940	0.1514
FPFH	0.1467	0.1586	0.2137	0.2219	0.2791	0.1339	0.0720
PPFH	0.6216	0.5808	0.5979	0.6205	0.6026	0.5747	0.3029
SHOT	0.1512	0.1497	0.1862	0.1854	0.2205	0.1671	0.1045
TOLDI	0.4043	0.3596	0.4202	0.4429	0.4481	0.3770	0.2681
HoPPF	0.6996	0.6248	0.6495	0.6496	0.6605	0.5186	0.4143
PPTFH	**0.7470**	**0.6895**	**0.7295**	**0.7501**	**0.7508**	**0.6487**	**0.4538**

The best ${\mathrm{AUC}}_{pr}$ results are in bold fonts.

**Table 6:** The best surface matching performance ($F_{1}$ scores) for different datasets.

Datasets	Bologna (0.9 mr Noise + 1/4 Downsample)	UWA (1/4 Downsample)	SDSR	Kinect
Method				
PFH	0.7424	0.7529	0.8972	0.5652
FPFH	0.7519	0.7594	0.8889	0.6739
PPFH	0.7159	0.9260	0.8704	0.6304
SHOT	0.9104	0.8272	0.8889	**0.8696**
TOLDI	0.9008	0.9111	0.8889	0.8261
HoPPF	0.8507	0.9322	0.9074	0.6304
PPTFH	**0.9123**	**0.9511**	**0.9630**	**0.8696**

The best $F_{1}$ scores results are in bold fonts.

**Table 7:** Pairwise rough registration results using the proposed PPTFH descriptor on the WHU-TLS datasets.

Data	Mountain	Campus	Residence	Riverbank	Heritage building	Total
Correct/total	5/5	8/9	5/6	6/6	8/8	32/34
Precision	100%	88.89%	83.33%	100%	100%	94.12%

## Data Availability

Not applicable.
